# Supplementary material for: Guided-deconvolution for correlative light and electron microscopy
Source: PLoS One. 2023 Mar 9;18(3):e0282803. doi: 10.1371/journal.pone.0282803 (PMC9997956; doi:10.1371/journal.pone.0282803)
Supplement: S1 Fig — a) The restoration becomes dim if λ ≃ ε. It yields in missing information of the structures in high-frequency. b) The algorithm will not very well restore the intensity distribution if λ < ε. c) The weight is too much on the EM-image which led to an incomplete removal (red arrow) of the non-fluorescent spokes if λ > > ε. d) The EM guidance will not contribute if λ is too small. The IG deconvolution generates good results if λ > ε (see Fig 1e). f) The NCC curves when the parameters are selected the same as in this figure. (PDF) [file pone.0282803.s001.pdf]

SI Fig 1

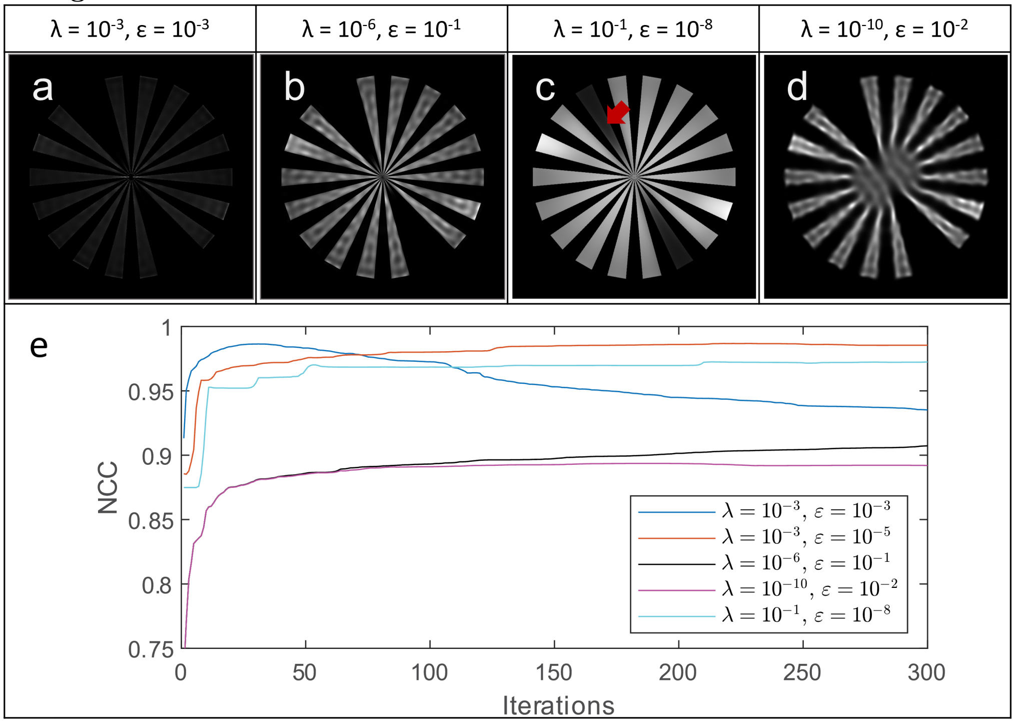

Restorations of IG deconvolution with different combination of parameters. a) The restoration becomes dim if $\lambda \simeq \varepsilon$. It yields in missing information of the structures in high-frequency. b) The algorithm will not very well restore the intensity distribution if $\lambda < \varepsilon$. c) The weight is too much on the EM-image which led to an incomplete removal (red arrow) of the non-fluorescent spokes if $\lambda >> \varepsilon$. d) The EM guidance will not contribute if λ is too small. The IG deconvolution generates good results if $\lambda > \varepsilon$ (see Figure 1e). e) The NCC curves when the parameters are selected the same as in this figure.
