# Supplementary material for: Guided-deconvolution for correlative light and electron microscopy
Source: PLoS One. 2023 Mar 9;18(3):e0282803. doi: 10.1371/journal.pone.0282803 (PMC9997956; doi:10.1371/journal.pone.0282803)
Supplement: S2 Fig — At low IG strength, a) TV regularization dominates. b) Balanced TV and IG strength yields good recovery of both, functional and morphology information. c) Strong weights of IG over emphasize the EM structural detail which overwrites the functional information. (PDF) [file pone.0282803.s002.pdf]

SI Fig 2

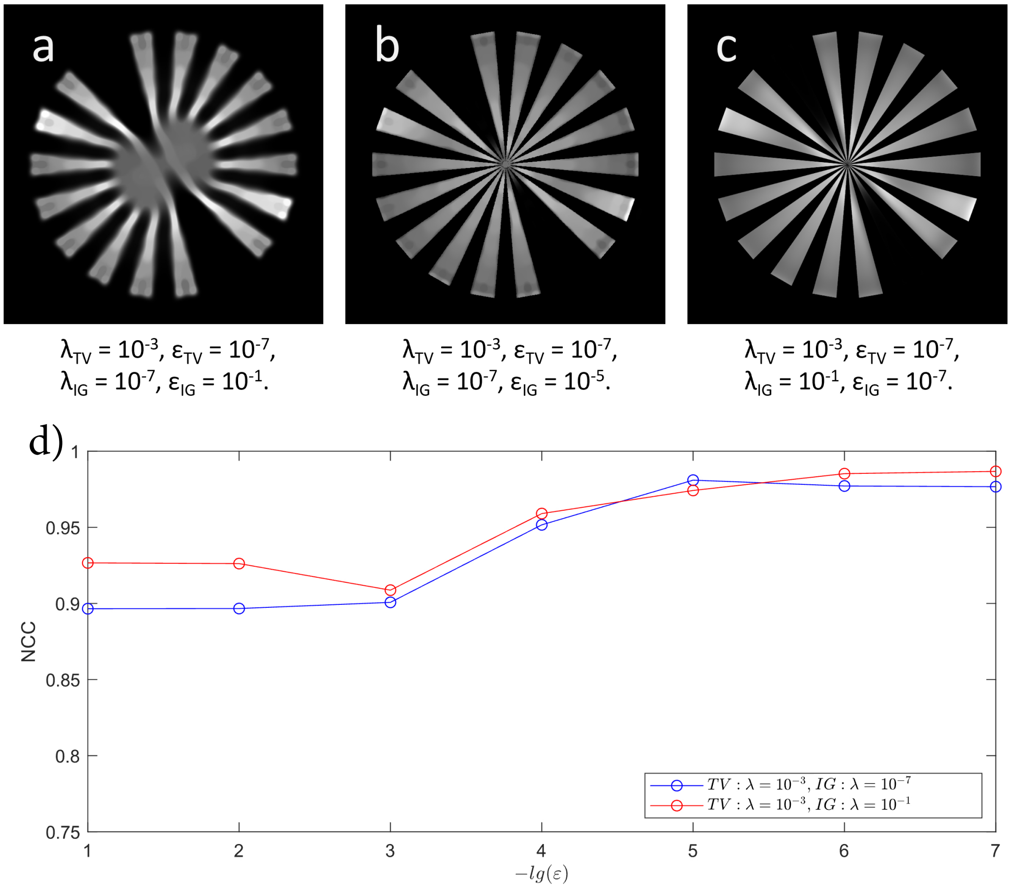

Restorations of TV & IG method at varying strength if the IG part. At low IG strength, a) TV regularization dominates. b) Balanced TV and IG strength yields good recovery of both, functional and morphology information. c) Strong weights of IG over emphasize the EM structural detail which overwrites the functional information. d) quality comparison for different influence of IG and **ε**.
