# Supplementary material for: Guided-deconvolution for correlative light and electron microscopy
Source: PLoS One. 2023 Mar 9;18(3):e0282803. doi: 10.1371/journal.pone.0282803 (PMC9997956; doi:10.1371/journal.pone.0282803)
Supplement: S3 Fig — The images in the left column are the restorations with the same λ at various ε. The images in the top row are the restorations with the same ε but at different λ. The final restoration of the EG deconvolution is less dependent on the parameters. Unless λ is too large, which enforces the EM information too much, the other restored images are very similar to the ground truth in terms of NCC. A larger λ can lead to faster convergence. The influence of ε is so small that there is no perceivable difference to the final result as supported by overlapping curves. (PDF) [file pone.0282803.s003.pdf]

SI Fig 3

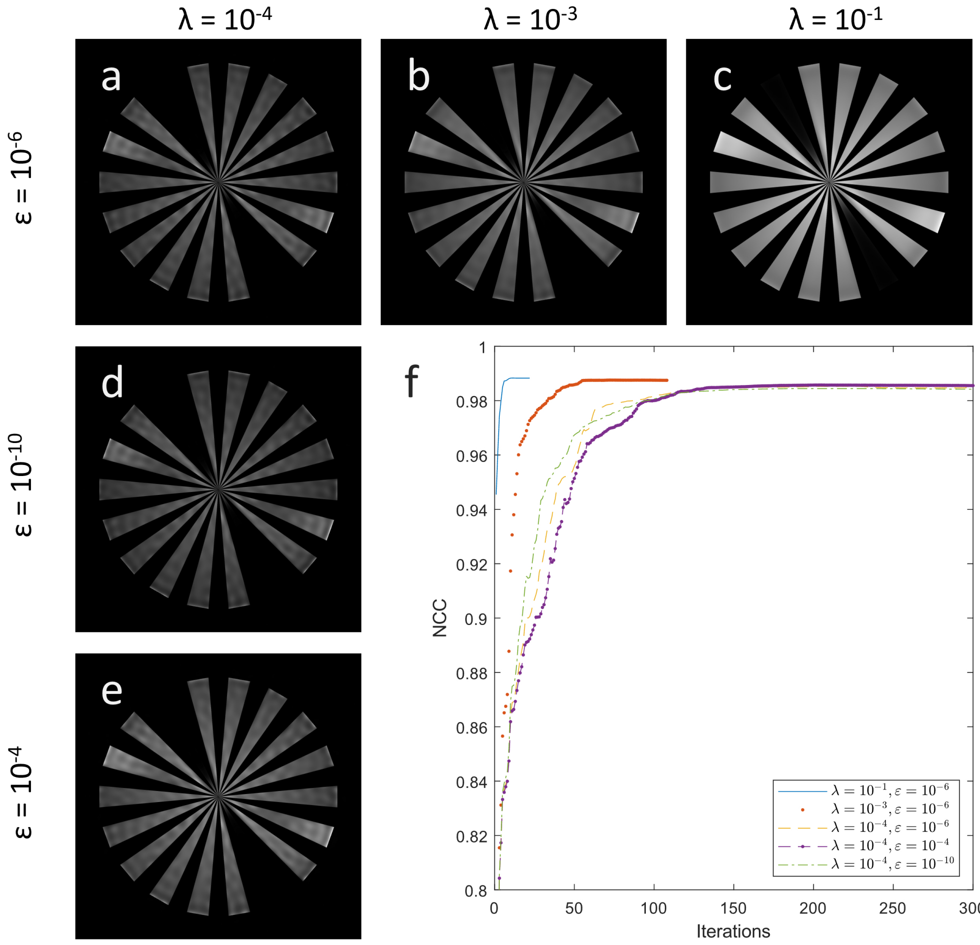

EG deconvolution using various parameters. The images in the left column (a,d,e) are restorations with the same λ at various ε. The images in the top row (a,b,c) are restorations with the same ε but at different λ. The EG deconvolution quality is less dependent on the parameters. f) reconstruction quality comparison. Unless λ is too large, which overly enforces EM information, the restored images are very similar to the ground truth in terms of NCC. A larger λ can lead to faster convergence. The influence of ε is so small that there is no perceivable difference as supported by overlapping curves.
