# Supplementary material for: Guided-deconvolution for correlative light and electron microscopy
Source: PLoS One. 2023 Mar 9;18(3):e0282803. doi: 10.1371/journal.pone.0282803 (PMC9997956; doi:10.1371/journal.pone.0282803)
Supplement: S4 Fig — a) The gradient guidance. b) The restored image only reconstruct good low-frequency structures if λ is slightly smaller than ε. c) The algorithm will not do restoration if λ ≥ ε. d) The EM information will not provide sufficient strength for the guidance if λ is too small. the best restoration happens when λ < ε (see also Fig 1h). (PDF) [file pone.0282803.s004.pdf]

SI Fig 4

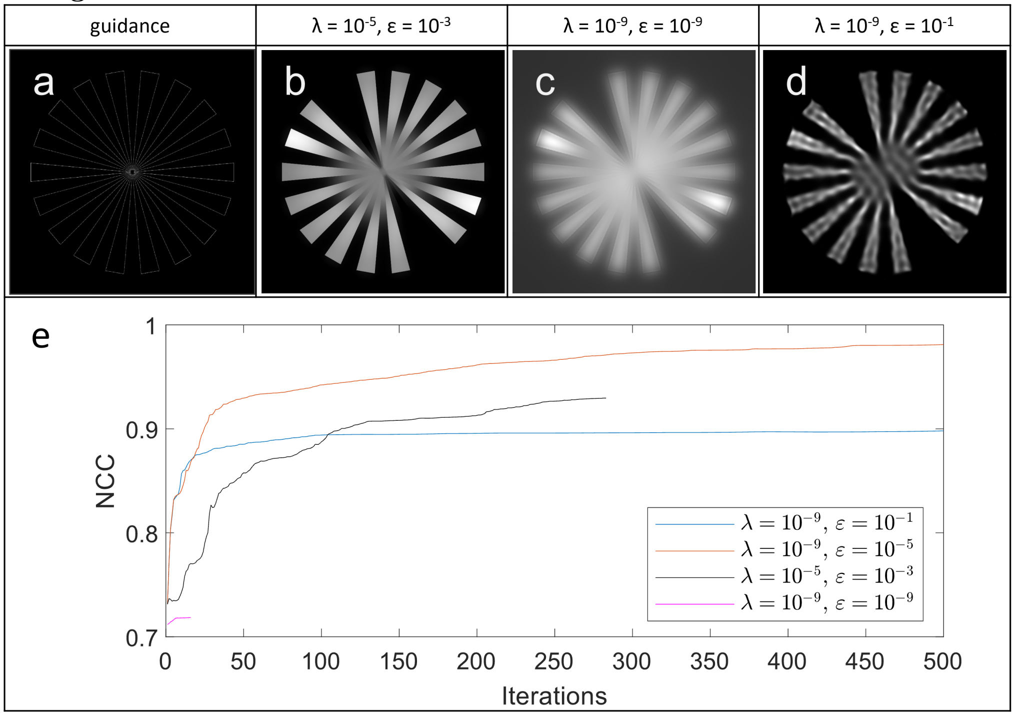

Restorations of GG deconvolution at different parameter settings. a) The gradient guidance. b) The restored image only reconstruct good low-frequency structures if λ is slightly smaller than ε. c) The algorithm will not do restoration if $\lambda \geq \varepsilon$. d) The EM information will not provide sufficient strength for the guidance if λ is too small. the best restoration happens when $\lambda < \varepsilon$ (see also Fig. 1h).
