# Supplementary material for: Guided-deconvolution for correlative light and electron microscopy
Source: PLoS One. 2023 Mar 9;18(3):e0282803. doi: 10.1371/journal.pone.0282803 (PMC9997956; doi:10.1371/journal.pone.0282803)
Supplement: S5 Fig — a) The preprocessed EM images. b) The GG restored images (λGG = 10−9, εGG = 10−4, λTikhonov = 10−8). c) The overlay of the restored images and the EM images. The preprocessing of the EM image was based on the Isodata algorithm [26]e. The result in the bottom row was generated when the EM image was trained in 3 classes with the Weka segmentation in Fiji. (PDF) [file pone.0282803.s005.pdf]

SI Fig 5

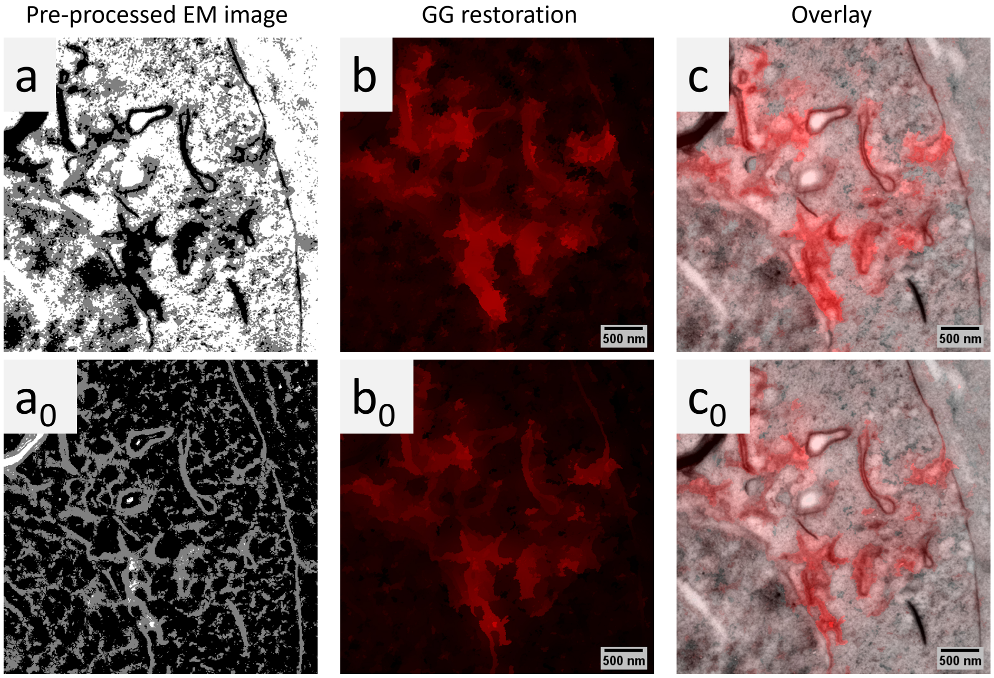

Restored images of GG deconvolution with different EM guidance. a) The pre-processed EM image. b) The GG restored image ($\lambda_{\mathrm{GG}} = 10^{-9}$, $\varepsilon_{\mathrm{GG}} = 10^{-4}$, $\lambda_{\mathrm{Tikhonov}} = 10^{-8}$). c) The overlay of the restored image and the EM image. The pre-processing of the EM image was based on the Isodata algorithm [26]. The result in the bottom row (a0, b0, c0) was generated segmenting the EM image into 3 classes with the Weka segmentation in Fiji.
