# Supplementary material for: Guided-deconvolution for correlative light and electron microscopy
Source: PLoS One. 2023 Mar 9;18(3):e0282803. doi: 10.1371/journal.pone.0282803 (PMC9997956; doi:10.1371/journal.pone.0282803)
Supplement: S1 Methods — (PDF) [file pone.0282803.s007.pdf]

SI Fluorescence beads sample preparation and images acquisition The sample solution was a mixture of orange (ThermoFisher Scientific, F8794, 565 nm /580 nm) and red (ThermoFisher Scientific, T8870, 633 nm /720 nm) fluorescence beads. The original solutions were diluted with a ratio $(1 : 10^4)$ in water. The diluted solution was then mixed in a ratio of 1:1. Drop 5 μl solution on a Formvar coated TEM grid; Poly-L-Lysin was used to attach the beads to the film and ensure a sufficient distribution of the particles. After 2 minutes, the rest of the solution was removed with a paper filter (Whatman No. 1). After completely drying, the TEM grid was mounted on the cover glass with glycerin. After the light microscopy image acquisition, the TEM grid was removed from the cover glass. The TEM grid was then washed with water, such that the glycerine was removed from the TEM grid. At the end the sample was imaged by a transmission electron microscope.

The fluorescence microscopy images were acquired by a confocal laser scanning microscope LSM980 with an 40$\times$ water objective. The sample was excited by 561 nm and 639 nm lasers. The refractive index was 1.33 and the NA was 1.2. The emission wavelength of orange (red) beads is 603 nm (719 nm).

TEM imaging was performed on a FEI Tecnai G^2 20 (ThermoFisher Scientific) at an accelleration voltage of 200 kV. Images were recorded on a Megaview CCD camera (1376 $\times$ 1024 image pixels, Olympus Soft Imaging Solutions, OSIS). Image intensity was adjusted to yield a max. of approximately 1000 counts.
